# Exploring the implementation and integration of structured medication reviews in primary care: a qualitative evaluation using normalisation process theory

**DOI:** 10.3399/BJGP.2025.0522

**Published:** 2026-07-14

**Authors:** Claire Reidy, Anna Seeley, Katherine Tucker, Adaku Agwunobi, Paul A Bateman, Christopher Elles Clark, Andrew Paul Clegg, Gary A Ford, Seema Gadhia, William Hinton, FD Richard Hobbs, Sundus Jawad, Kamlesh Khunti, Gregory YH Lip, Simon de Lusignan, Jonathan Mant, Deborah McCahon, Bernardo Meza-Torres, Rupert A Payne, Rafael Perera-Salazar, Samuel Seidu, James Sheppard, Marney Williams, Cynthia Wright Drakesmith, Richard J McManus, Rebecca K Barnes

**Affiliations:** 1 Nuffield Department of Primary Care Health Sciences, University of Oxford, Oxford, UK; 2 Saïd Business School, University of Oxford, Oxford, UK; 3 Exeter Collaboration for Academic Primary Care, University of Exeter, Exeter, UK; 4 Academic Unit for Ageing and Stroke Research, University of Leeds, Leeds, UK; 5 Bradford Institute for Health Research, Bradford Teaching Hospitals NHS Foundation Trust, Bradford, UK; 6 Radcliffe Department of Medicine, University of Oxford, Oxford, UK; 7 Oxford University Hospitals NHS Foundation Trust, Oxford, UK; 8 Health Innovation Oxford and Thames Valley, Oxford, UK; 9 NHS Frimley Integrated Care Board, Windsor, UK; 10 Diabetes Research Centre, University of Leicester, Leicester, UK; 11 Liverpool Centre for Cardiovascular Science, University of Liverpool, Liverpool, UK; 12 Liverpool John Moores University and Liverpool Heart & Chest Hospital, Liverpool, UK; 13 Danish Center for Health Services Research, Department of Clinical Medicine, Aalborg University, Aalborg, Denmark; 14 Clinical Department of Cardiology, Lipidology and Internal Medicine with Cardiac Intensive Care Unit, Medical University of Białystok, Białystok, Poland; 15 Primary Care Unit, Department of Public Health and Primary Care, University of Cambridge, Cambridge, UK; 16 Centre for Academic Primary Care, Population Health Sciences, Bristol Medical School, University of Bristol, Bristol, UK; 17 Brighton and Sussex Medical School, University of Brighton and University of Sussex, Brighton, UK

**Keywords:** clinical pharmacists, general practice, medicines optimisation, normalisation process theory, polypharmacy, structured medication reviews

## Abstract

**Background:**

Structured medication reviews (SMRs) were introduced into primary care in England in 2020 for patients living with multiple long-term conditions (MLTC), polypharmacy, increased frailty, in care homes, or at risk of medicines-related harm. SMRs aim to optimise the therapeutic potential of medication and reduce medicine-related harms through holistic reviews.

**Aim:**

To explore the day-to-day work being undertaken with, and by, clinical pharmacists to implement, embed, and integrate SMRs into practice, and consider how to optimise SMRs.

**Design and setting:**

Qualitative one-to-one interviews with clinical pharmacists undertaking SMRs and SMR service leaders/managers (SMR leads) in England between February 2023 and November 2024.

**Method:**

Participants were recruited as part of a wider evaluation of the roll-out of SMRs in England. Interview topic guides and qualitative data analysis were informed by normalisation process theory.

**Results:**

Eighteen clinical pharmacists and five SMR leads participated. Participants reported often having to explain the purpose of SMRs and clinical pharmacists' roles to patients, partly owing to patients not being informed about SMRs. Participants valued SMRs and expressed that trust-building and tailored consultations were important for optimising medications. Integration of SMRs into routine practice varied because of high workload, inconsistent leadership support, inadequate administrative/pharmacist technician resource, and lack of training. However, participants described SMRs as valuable for identifying and addressing unmet needs and supporting holistic, person-centred care across MLTC pathways.

**Conclusion:**

The findings demonstrate the need for improved information on SMRs for patients and primary care teams, adequate and appropriate resource allocation, and enhanced support for consultation skills training to optimise medicines use.

**How this fits in**

Structured medication reviews (SMRs) were formally introduced to primary care in 2020 to address the challenges of managing polypharmacy in an ageing population with patients who have increasingly complex needs and multiple long-term conditions. SMRs were introduced, alongside the expansion of clinical pharmacist roles in general practice, as a comprehensive, person-centred review of all patient’s medicines. This qualitative evaluation examined the day-to-day work of implementing and embedding SMRs, highlighting challenges to implementation and integration of SMRs into routine practice. Our findings reveal challenges to the sustainability of SMRs and identify opportunities for optimisation, including addressing pharmacists’ training needs and resource allocation for administrative and pharmacy technician support.

## Introduction

Medicines-related harm poses a significant burden on patients and the NHS. Adverse drug events, which include medication errors, adverse drug reactions, allergic reactions, and overdoses, are estimated to cost the NHS approximately £400 million annually in hospital admissions alone.^1^ Of the estimated 237 million medication errors occurring annually in England, nearly 40% take place in primary care and over 40% in care homes.^2^ Although prescribed medications play a vital role in improving health outcomes and prescribing multiple medications (‘polypharmacy’^3^) is often clinically necessary, older adults are more likely to be prescribed multiple medications^4^ and are more susceptible to harm from medicines.^5^ Such harm includes hospital admissions, falls, drug interactions, and mortality, especially for those living with frailty or multiple long-term conditions (MLTCs).^4–9^ Structured medication reviews (SMRs) have been introduced to help manage polypharmacy, particularly for patients with MLTCs.^10,11^

During 2020/2021, SMRs became a contractual requirement under the NHS England Network Contract Directed Enhanced Service (DES) for the then newly created primary care networks (PCNs) in England.^12^ The DES contract included delivering SMRs through appropriately trained healthcare professionals (primarily, clinical pharmacists).^13^ This aligned with the expansion of this role through the Additional Roles Reimbursement Scheme (ARRS) and the Investment and Impact Fund (IIF), a financial incentive scheme rewarding performance against national prescribing and prevention targets.^12^

SMRs were targeted at patients with severe frailty, in care homes, taking ≥10 medications, medicines associated with medication errors, or addictive medications.^13^ They offer a more comprehensive, structured, and holistic approach to medicines management,^14^ as opposed to prioritising efficiency over thoroughness.^15^ It was hoped that by providing individual and holistic reviews of medications, pharmacists could optimise medication use, aligned with patients’ needs and preferences.^13,16–20^

This evaluation explores the experiences of clinical pharmacists and SMR service leaders and managers a few years on from SMR roll-out. Informed by normalisation process theory (NPT),^21^ we focus on the everyday work undertaken by organisations and individuals to implement, embed, and integrate SMRs into general practice at a PCN and practice level, and to consider how SMRs might be optimised and sustained.

## Method

### Study design

Semi-structured qualitative interviews were undertaken as part of a wider mixed-methods evaluation (Optimising StruCtured medicAtion Reviews — OSCAR)^22–24^ to explore the implementation and roll-out of SMRs in England.

### Participant recruitment and sampling

We spoke to clinical pharmacists undertaking SMRs in primary care settings, and SMR service/medicines optimisation leaders and managers (referred to as ‘SMR leads’), working within different settings. Some participants held dual clinical and leadership roles. For participant recruitment, those who left their details in a survey of SMRs as part of the wider OSCAR evaluation^24^ were contacted, and others working in OSCAR case study sites were invited to participate by the research team via email. All participants gave written or digitally recorded informed consent.

### Data collection

All interviews were conducted by the fourth author (PhD, post-doctoral researcher), the second author (GP and primary care DPhil student), and the senior author (PhD, experienced qualitative researcher). Neither the fourth author nor the senior author had any prior relationship with the study participants. The second author had previously worked briefly with one of the participants but was not working with them at the time of interview. Interviews took place on Microsoft Teams or via telephone between February 2023 and November 2024.

Flexible topic guides (see Supplementary Information S1 and S2), sensitised to the constructs of NPT, were developed with input from clinicians and patients. Interviewees were asked to reflect on the different kinds of work required in preparing for, conducting, and implementing SMRs, including resources used and how different patient groups were prioritised. Data collection continued until no new insights regarding the research questions and theoretical framework were presented.^25^ All interviews were digitally recorded and professionally transcribed verbatim.

### Data analysis

#### Theoretical framework

NPT is a methodological framework that aids in exploring the implementation of healthcare practices as well as providing feedback to help sustain further implementation.^21^ In the context of SMRs, NPT considers the implementation, mechanisms, and the collaborative work involved in integrating SMRs into routine healthcare practice, in terms of personal and group resources.^26^ NPT can shed light on the unseen work involved in embedding SMRs within a healthcare system already challenged by competing demands.^27^ The four key NPT constructs are outlined in Box 1.

**Box 1.** Normalisation process theory (NPT) constructs

NPT constructs	Interpretation
**Coherence**	Addresses the question, ‘What is the work?’. It involves the sense-making processes that individuals and teams undertake to understand and define the purpose and tasks needed to integrate SMRs into routine practice
**Cognitive participation**	Asks ‘Who does the work?’ and explores how individuals and organisations engage with and commit to a new practice and considers buy-in and ongoing relational work
**Collective action**	Focuses on ‘How does the work get done?’ and highlights the operational activities needed to implement SMRs, and how these practices are integrated into workflows
**Reflexive monitoring**	Is essential for embedding and maintaining clinical practices. It represents how individuals and teams evaluate the impact and value of SMRs, and the continued refinement of SMRs

SMR = structured medication review.

Following transcription, interview records were anonymised and uploaded to NVivo (version 14). The data were coded both inductively, guided by Braun and Clarke’s^28^ six-step framework for thematic analysis, and deductively, using the primary NPT constructs and secondary constructs.^27^ First, the first author (an experienced primary healthcare researcher and with experience of NPT) became familiar with the data through multiple readings of a subset of transcripts, creating an initial list of ideas from the data. These steps created initial codes, which were then reviewed together with the senior author. This was followed by inductive and deductive development of broad themes within the NPT constructs. Emerging findings were presented to the wider study team for sense-checking and discussion. The wider study team comprised multiple members with varying clinical and research backgrounds, and patient and public involvement, enabling investigator triangulation.^29^

## Results

Interviews were conducted with 23 participants, 18 of whom were clinical pharmacists (or senior clinical pharmacists) and five were ‘SMR leads’ (see Table 1). Participants were from seven different NHS England regions and interviews lasted between 26 and 65 min (average 37 min). Informed by NPT, the themes identified are summarised in Box 2.

**Table 1.** Participant characteristics (*N* = 23)

Characteristic	*n* (%)^a^
**Age, years**	
20–29	3 (13)
30–39	11 (48)
40–49	7 (30)
50–59	2 (9)
**Gender**	
Female	15 (65)
Male	8 (35)
**Ethnicity**	
British South Asian	11 (48)
White British	7 (30)
Black African	2 (9)
Vietnamese/Chinese	1 (4)
Mixed British	1 (4)
White Other	1 (4)
**Location**	
North West	5 (22)
East Midlands	4 (17)
West Midlands	2 (9)
London	2 (9)
South Central	5 (22)
South East	3 (13)
South West	2 (9)
**Role**	
PCN-based clinical pharmacist	10 (43)
GP practice-based clinical pharmacist	4 (17)
Senior or lead pharmacist	4 (17)
PCN pharmacy lead	2 (9)
Social and research lead GP partner	1 (4)
Care homes medicines optimisation lead	1 (4)
Pharmacy lead	1 (4)
**Employer**	
PCN	11 (48)
GP practice	7 (30)
Community interest company	2 (9)
NHS England and PCN	1 (4)
NHS England and ICB	1 (4)
ICB	1 (4)
**Independent prescriber**	
Yes^b^	20 (87)
No	3 (13)
**Years qualified as a pharmacist, mean (SD) range**	13.65 (7.2) 2–26
**Years experience of conducting SMRs, mean (SD) range**	3.9 (3.7) 0.5–20^c^

a Unless otherwise stated. ^b^ *n* = 19 clinical pharmacists and *n* = 1 GP. ^c^SMRs were put into practice in 2020 but some pharmacists described doing what they considered to be SMRs before this time. ICB = integrated care board. PCN = primary care network. SD = standard deviation. SMR = structured medication review.

**Box 2.** Themes by normalisation process theory (NPT) constructs

NPT construct	Theme	
**Coherence**	Patient understanding of an SMR	
	Setting patient expectations for SMR consultations	
	Differentiating SMRs from other medication reviews	
**Cognitive participation**	Professional identity and role alignment	
	Organisational and peer engagement	
	Building legitimacy and patient trust	
**Collective action**	Patient identification and prioritisation for SMRs	
	Preparatory work for SMRs	
	Modes and settings of SMRs	
	SMR structure and process	
	Time allocation and workload implications	
	Interprofessional relationships	
	Training needs and approaches	Training needs
		Training approaches
	Conducting SMRs in care homes	
**Reflexive monitoring**	Identifying unmet patient needs and prescribing savings through SMRs	
	Positioning SMRs within MLTC management pathways	

MLTC = multiple long-term condition. SMR = structured medication review.

### Coherence

#### Patient understanding of an SMR

Participants often highlighted the necessity of explaining the role and purpose of an SMR to patients. They remarked that many patients still had difficulty differentiating between community pharmacists and clinical pharmacists working in primary care. This meant that clinical pharmacists often had to explain their role and how it differed:

*‘And most of them will say, “I didn't know they had a pharmacist attached to the practice”. And then often you have to say to them, “No, I’m not from your local pharmacy”, because once you say pharmacy they think you’re from the community pharmacy.’* (Pharmacist [PHR] 10, qualified 19 years, SMRs 6 years, independent prescriber [IP])

It was reported that, potentially because of a lack of understanding, some patients viewed the SMR as a procedural exercise to continue access to repeat medications. Some interviewees supposed that providing patients with more information about SMRs beforehand would improve the value attributed to SMRs.

#### Setting patient expectations for SMR consultations

Some practices offered patients booked appointments for SMRs in advance whereas others did not, which participants described as disconcerting for patients. Most of our participants wanted to give patients information and time to prepare, which could reduce time spent explaining the purpose of SMRs and allow greater focus on medication needs and queries:

*‘... you do really need to get somebody an appointment because I want them to anticipate it so they can think about any questions as well … cold calling … I don’t think that’s the best approach.’* (PHR 16, qualified 24 years, SMRs 6 years, IP)

#### Differentiating SMRs from other medication reviews

Many of the pharmacists spoke of SMRs as holistic reviews, which provide the potential to prescribe better for patients. They referred to SMRs as more complex and thorough than GP-led medication reviews, yet expressed that other clinicians sometimes lacked this comprehension:

*‘I think it’s a skill thing, you ask a pharmacist what a SMR is and you’ll get a completely different story to what a GP says … So some GPs will see it as a tick box. “I’ve had a look through the medicines and yeah … I’ve done a SMR” …* [but] *you didn't actually speak to them.’* (PHR 05, qualified 26 years, SMRs 4 years, IP)

### Cognitive participation

#### Professional identity and role alignment

Those interviewed were confident in the role of clinical pharmacists to appropriately and thoroughly review multiple medications in-depth across several conditions. Some described how they were able to pick up on different and additional details to GPs, and many had extensive experience in relevant clinical specialities, such as frailty:

*‘But yeah, I mean, it, it’s stuff that we, we do all the time, isn’t it. As pharmacists we talk about medicines. It’s sort of our bread and butter, really.’* (PHR 01, qualified 7 years, SMRs 3.5 years, IP)

Interviewees also spoke of holistic SMRs as useful touchpoints for pharmacists to engage with and manage MLTCs:

*‘... you’re saying, asthma is this part … come back next week and we’ll do your diabetes … it feels like we’re chopping a person up into their little conditions. Whereas … their health is a holistic thing.’* (PHR 01, qualified 7 years, SMRs 3.5 years, IP)

#### Organisational and peer engagement

Some interviewees described a lack of senior buy-in, resulting in inadequate resources for SMRs (for example, costing in pharmacist technicians, administrative support, or enough pharmacist time), making it difficult to see most patients who could benefit from one. One SMR lead spoke of working with the PCN clinical director and PCN board to negotiate pharmacist time allocated to SMRs:

*‘We looked …* [at] *how many people could be eligible for an SMR in line with what the contract says … 45 000 people … across three practices … the challenge is where you start to really stratify that … also what percentage of the, the whole-time equivalent that pharmacists have … versus every other thing they do.’* (SMR lead [LDR] 03, qualified 12 years, SMRs 3 years)

Where adequate resources had been allocated, interviewees reported being able to appropriately tailor their week (for example, holding weekly SMR clinics, booking patients in, and arranging/checking blood tests). They could then focus their time better.

Many participants suggested that a lack of engagement and involvement from other clinicians was perhaps because of the lack of understanding of the purpose and value of SMRs. However, more established pharmacists described building good rapport with colleagues in their practice and improving appreciation of the length and breadth of SMRs, over time. This was especially relevant for PCN-based pharmacists who covered several practices.

#### Building legitimacy and patient trust

Those interviewed described how patients often appeared to view GPs as the primary, and sometimes sole, legitimate role for discussing medication decisions. When pharmacists initiated discussions around medication changes, this expectation was thought to undermine patient receptivity, requiring pharmacists to undertake additional relational work to establish legitimacy and trust:

*‘Sometimes when they* [patients] *hear … that you’re a pharmacist and trying to make changes to their medication they sometimes aren’t really too susceptible to it because they are, “why isn’t the GP doing it and why hasn’t the GP noticed that before?”’* (PHR 07, qualified 2 years, SMRs 2 years)

Interviewees suggested that patients’ understanding of the pharmacist role and purpose of SMRs was central to cognitive participation, shaping how openly patients discussed their medications and whether they felt able or willing to consider changes to their existing medications.

### Collective action

#### Patient identification and prioritisation for SMRs

Interviewees described undertaking SMRs with patients from DES contract priority cohorts, with some additionally targeting specific medication combinations or potentially harmful omissions (that is, instances where appropriate medicines had not been prescribed or continued, creating potential risk) outlined in the 2022/2023 IIF. Patients were commonly identified through proactive, automated searches of the practice’s electronic health records (via Ardens, Eclipse, or EMIS), with others identified opportunistically. These systems worked well when clear criteria for patient prioritisation were in place; however, after the withdrawal of IIF incentives to undertake SMRs, prioritisation became more variable. Although DES and IIF guidance provided a national framework for selection, decisions about which patients were reviewed were also shaped by local organisational capacity, pharmacist time, leadership engagement, and practice- or PCN-level strategic priorities.

#### Preparatory work for SMRs

Interviewees described undertaking extensive preparatory work to ‘build a picture’ before speaking to patients. This involved determining what condition(s) the patient had and understanding what was going on in their lives medically (through hospital letters, reported medication changes, blood tests, including liver function, cholesterol tests, and blood pressure readings). Also, assessment of medication prescribing patterns (such as what is taken and when requested, if at all) and inappropriate combinations. Preparatory work enabled better understanding of the complexity of the patient’s needs and history of the patient.

This preparation could take 10–20 min and aimed to optimise and focus SMR time. It was also noted that this work reduced over time as pharmacists knew the patients better (so needed less preparatory work) and patients were more prepared.

Importantly, however, blood test results were often not undertaken before an SMR, which made initiating changes during an SMR difficult or impossible, and so follow-up appointments were required to initiate, or even consider, some changes.

#### Modes and settings of SMRs

SMRs were undertaken in the practice, care homes or patients’ homes, or remotely. The majority were conducted remotely by telephone because of time efficiencies or lack of clinic space. Face-to-face SMRs were preferred for some patients who were older or with more complex needs (especially to assess frailty), or for patients with English-language difficulties, hearing difficulties, or when others (such as carers/family members) were involved. Face-to-face SMRs were described as better at determining whether patients understand their medications, for example, by having access to facial expressions and other non-verbal cues:

*‘I would prefer a face-to-face SMR where patients come in, bring in all their medication with them ...* [and] *understanding of their body language,* [and] *whether they take their medication appropriately.’* (PHR 03, qualified 30 years, SMRs 3 years, IP)

Several participants noted that SMRs were introduced during the COVID-19 pandemic, coinciding with rapid service redesign, remote working, and significant organisational strain. This context shaped early implementation and was perceived to have ongoing implications for workload pressures and reliance on remote consultations.

#### SMR structure and process

After establishing the purpose of the appointment, interviewees described going through patients’ test results (if available) and medications one by one, often while following a clinical consultation guide/‘template’ (for example, a multimorbidity, self-made, or generic electronic template). Templates were used as a prompt and to initiate broader, open questions, such as to understand how medication is taken and any side effects experienced. Interviewees reported exploring social circumstances and questions or concerns about the medication or other health matters. Then coming to a shared agreement about any changes (for example, medication doses, adding, or removing medication). Where changes were agreed, these sometimes resulted in further follow-up (especially if dependent on blood tests):

*‘I have my own set of questions … have you got a good routine for your medication? Any over the counter medication … we normally go through all each and every single medication. We go through their concerns first …* [and] *try to come to sort of shared agreement with regards to a plan … that can result in another follow-up appointment.’* (PHR 07, qualified 2 years, SMRs 2 years)

Although existing templates were useful to aid the process of SMRs, they were also described as detailed and sometimes difficult to navigate in real-time consultations, particularly for those newer to the role.

#### Time allocation and workload implications

SMRs were typically described as lasting at least 20 min, and often extended to 30 or 45 min or more, depending on the complexity of patients’ needs. Some also required follow-up consultation(s). The time allocated for SMRs, compared with standard GP appointments, was seen as crucial in building rapport and trust, and allowing deeper exploration of the complexity of patients’ needs. Interviewees said this time enabled patients to reflect on their medication use and to speak openly, providing more opportunity for partnership and shared decision making:

*‘There was a lot of to-ing and fro-ing about how long we were gonna be allowed to do them in … so I stood my ground and still kept half an hour for an SMR plus 20 minutes follow-up* [if needed] *… in some areas pharmacists have ended up doing them in a lot shorter time, that doesn't give the patient as much freedom I think to actually be honest with you and kind of get that rapport built.’* (LDR 04, qualified 14 years, SMRs 3 years)

Giving patients sufficient time to express their concerns was frequently described as essential to conducting an SMR well, ensuring that patients’ perspectives were being heard and addressed.

#### Interprofessional relationships

Where interviewees did not have the authority or confidence to change an existing medication or start a new one (for example, not an IP or unfamiliar with a particular medication/condition), it was resolved with a GP. This relied on collaborative relationships with GPs in the practice.

#### Training needs and approaches

There were two sub-themes, training needs and training approaches.

In terms of training needs, interviewees appeared to be familiar and comfortable with making changes with medications that matched their own professional knowledge and experience. However, some changes were reported as being difficult, largely concerning pain and addictive medications. Any dose changes here were depicted as a slow and difficult process, requiring careful conversations, advanced consultation skills, and multiple follow-ups:

*‘It’s resistance to people wanting to bring themselves off pain relief medication and … I mean refusing tablet doses to be reduced. So, despite being in their best interests, they see it differently … so it’s a question of understanding and educating them and that can be potentially problematic as well.’* (PHR 06, qualified 10 years, SMRs 3.5 years, IP)

Although pharmacists expressed that conducting SMRs aligned well with their professional role, participants identified ongoing training needs, particularly for newer clinicians, and to optimise SMRs. These included developing expertise in managing specific conditions or medicines and in engaging in difficult conversations, especially around deprescribing, which was widely described as one of the most relationally challenging aspects of SMRs.

Templates were seen as particularly helpful for less experienced pharmacists, who were less confident in conducting complex, holistic reviews. The templates functioned as a safety net to ensure important domains were not missed. However, some more experienced pharmacists described using templates more flexibly over time, adapting them to support a more conversational and patient-led approach.

Interviewees commonly highlighted training needs, often centred around building confidence and consultation skills for stopping or reducing certain medications. These were particularly around building rapport and trust with patients, encouraging open conversations, and supporting patient disclosure. Additional priorities included shared decision making, interpreting non-verbal cues, and developing knowledge and confidence in deprescribing for patients in care homes owing to deteriorating physiology and increased frailty. Mental health skills and consultation length and structure were also noted.

In terms of training approaches, interviewees described the training they had undertaken to prepare them for conducting SMRs (see Box 3). There was huge variation in the types of training described (which largely depended on the PCN or practice) as well as the time allocated to receive training.

**Box 3.** Training approaches described by interviewees

**Training approach**	**Description**
**Shadowing**	Shadowing a more senior and experienced clinician gave insight into how consultations were conducted, providing a model of how to carry them out, especially for patients with more complex cases, how templates are used, and use of clinical systems. They provided examples of more straightforward SMRs, leading up to more complex ones.
**Leaders providing monthly support and training sessions**	One PCN leader described giving support once a month to clinical pharmacists undertaking SMRs for the first time. They gave them opportunities to practice speaking with patients about medicines and sitting with them while they undertook SMRs. They also encouraged clinical pharmacists to come back and ask further questions, building their skills gradually, to support patients with more complex cases.
**Specific SMR training — Centre for Pharmacy Postgraduate Education pathway**	One interviewee mentioned the Centre for Pharmacy Postgraduate Education pathway that covered their first 18 months in primary care, with training in medical conditions and SMR consultations through workshops and case studies
**Creating communities of practice**	One PCN lead spoke of the creation of a community of practice of those with a shared interest from different disciplines (such as GPs, clinical pharmacists, practice nurses, and commissioners) in *‘addressing and tackling problematic polypharmacy’*, and where opioids are discussed
**Mentoring and supervision**	Some spoke of working with others to train and build confidence to undertake SMRs, and watching others do SMRs, especially with potentially more difficult scenarios, such as supporting patients to reduce and stop opioids. One PCN provided a mentorship scheme, with training and webinars to support clinical pharmacists undertaking SMRs, providing a place to answer queries and build expertise, confidence, and knowledge.
**SMR training day**	With the aim of growing clinical knowledge, as well as the confidence of GPs in the PCN
**Workshops**	Workshops delivered nationally, such as on managing opioid medications

PCN = primary care network. SMR = structured medical review.

#### Conducting SMRs in care homes

Care home SMRs were widely perceived as resource intensive. The complexity of patients’ cases, as many have significant cognitive impairment, along with the need to engage multiple parties (including patients, families, carers, and specialist staff), was considered challenging.

There was considerable variability in the management of SMRs in care homes. In some areas, GPs were designated to manage care home residents because of the complexities of these patients’ cases. Some PCNs assigned pharmacists to specific care homes, designated particular care homes to individual practices, or a single practice took responsibility for all care homes within the PCN.

One barrier was limited WiFi in care homes, which obstructed access to patient records. To facilitate the administration of SMRs, some care homes allocated tasks (such as logging patients’ blood pressure/weight) to night staff, to copy and paste into relevant records. Medication changes were often reported as well-managed within care home protocols, particularly when aligned with their monthly medication cycles. When pharmacists engaged with and aligned with these procedures and protocols, and were familiar with care home staff, SMRs ran more smoothly.

### Reflexive monitoring

#### Identifying unmet patient needs and prescribing savings through SMRs

Two SMR leads described how they carried out local service evaluations or audits of their SMRs, with evidence that SMRs facilitated prescribing savings for the practice, the identification of work to help patients, and prevented hospital admissions:

*‘I’ve done a bit of a like a service evaluation … it’s led to some savings, but generally it’s led to some kind of intervention … It identifies work that we could, we needed to do … quite a few of the GPs … have said ...* [it’s] *what they were hoping for, not that they would do less, but the things that they were missing would get picked up.’* (LDR 02, qualified 19 years, SMRs 3 years, IP)

*‘When we audited our own SMRs … we were showing a* [prescribing] *savings of around £280* [per year] *per patient …* [and] *if you found the same problem in ten people … every ten you've prevented one hospital admission …* [but] *we are quite an experienced team.’* (LDR 05, qualified 23 years, SMRs 20 years, IP)

Interviewees described how SMRs have helped pick up patient care that has previously been missed, and led to interventions that would otherwise not have occurred. For example, SMRs have supported the identification of overlooked/misattributed clinical issues, review of long-neglected medications, optimisation of symptom management, and reduced medication burden and drug interactions. Further, establishing trust, continuity, and holistic care enabled them to support patients, such as those with complex pain, through safe, gradual medication reductions.

#### Positioning SMRs within MLTC management pathways

Interviewees saw SMRs as an important element of MLTC management, providing a holistic and patient-centred approach to reviewing medications while addressing broader aspects of care. SMRs allowed assessment of advice previously provided and clarification on whether patients fully understood medication guidance, while agreeing on strategies moving forward. SMRs also provided an opportunity to consolidate primary and secondary care medication management plans, supporting patients to feel more informed and have clearer understanding of their medications:

*‘We’ve just incorporated* [SMRs] *as part of our … Chronic Disease Process because that is the backbone as a pharmacy team of what we do … I think it just comes down to SMRs are really important. They are really good holistic reviews and they just support a really good prescribing process.’* (PHR 13, qualified 11 years, SMRs 3 years, IP)

## Discussion

### Summary

In exploring how SMRs were implemented and embedded into routine primary care, interviewees reported that patients’ understanding of SMRs and the clinical pharmacist’s role was often limited, with ‘cold calling’ and lack of preparation for patients hindering meaningful engagement. Pharmacists described strong professional identity and confidence in delivering SMRs, particularly when organisational support and resources (such as pharmacy technician input for blood test booking and preparation) were available. Trust-building with patients and clarifying the clinical pharmacist’s role were crucial for fostering engagement, as was relationship-building within practice teams, especially for PCN-based pharmacists working across multiple practices.

SMRs involved substantial operational work beyond the consultation itself, including preparation, follow-up, and coordination, and required adequate time, clinical expertise, and communication skills, particularly for deprescribing, patients with complex cases, and care home residents. However, variability in workflows, training, and resource allocation led to inconsistent integration across practices. Where SMRs were supported by protected time, administrative infrastructure, and alignment with chronic disease pathways, integration appeared more secure; where layered onto already stretched systems, sustainability was more fragile. From an NPT perspective, this highlights the ‘collective action’ required to embed SMRs into everyday practice.

Service leaders and managers reported using local audits and evaluations to assess the impact, identifying benefits such as uncovering and addressing unmet patient needs, timely interventions, prescribing cost savings, supporting MLTC management, and reductions in hospital admissions. Overall, SMRs were viewed as valuable opportunities to enhance patient engagement, clarify care plans, improve patient understanding, and improve medicines optimisation.

### Strengths and limitations

Using NPT enabled insights into the administrative, clinical, and relational work pharmacists undertake in implementing and conducting SMRs. It also illuminated how this work is shaped by evolving policy, resource constraints, and competing priorities. The study included a diverse sample of participants, varying in age, sex, ethnicity (both closely aligning with national figures^30^), role, experience, and geography. This heterogeneity enhanced the breadth of perspectives captured. However, most interviewees were IPs with several years of experience, which may limit the transferability of findings to less experienced pharmacists.

Despite this, there was strong consensus around training needs, particularly regarding consultation skills. Given the collaborative nature of SMR delivery, perspectives from GPs, practice managers, pharmacy technicians, and administrative staff could have further enriched understanding of interprofessional dynamics, buy-in, and relational and operational challenges. Trustworthiness and credibility of the findings were enhanced through investigator triangulation, regular team discussions, and involving stakeholders in study design and interpretation. Finally, the findings reflect a more mature phase of roll-out and a national programme that continues to evolve in a constantly shifting policy and operational context, and so continued evaluation as the landscape changes is recommended.

### Comparison with existing literature

Our findings reflect broader evidence on workforce planning in primary care, particularly the importance of structural and organisational support in embedding new clinical roles.^31,32^ Although the integration of clinical pharmacists has been widely studied,^31–34^ persistent challenges remain, most notably around role clarity, team collaboration, funding security, and demonstrating economic value.^33^ IP status is particularly valued by patients, and GPs are more likely to be supportive when they have experience working alongside pharmacists.^34^ Building trust with colleagues, patients, and carers is therefore crucial, as is access to appropriate training, sustained funding, and clear models of integration.^32,34,35^

In the context of SMRs, existing literature has shown a similarly mixed picture. Roll-out has been inconsistent, with pharmacists already embedded in practices adapting more easily, and newer pharmacists often relying on templates and established habits.^36^ These findings echo our own, especially around the tensions pharmacists face in collaborative working, time constraints, and balancing guidelines with individual patient needs, particularly in deprescribing.^36,37^ Managing medication changes in polypharmacy is often a process of ongoing adjustments and relational work, rather than one-off interventions,^37^ and requires preparatory and follow-up work.

Training is a recurring theme, with previous research noting that SMR training tends to emphasise clinical knowledge but offers limited feedback and support on consultation skills.^38^ Our findings confirm that although training is delivered in various ways, its quality and focus are inconsistent across regions. There remains a need to strengthen consultation skills for holistic, person-centred care.

### Implications for research and practice

SMRs were introduced during the COVID-19 pandemic, a period of rapid service reconfiguration, remote working, and workforce strain. This may have shaped the ways in which SMRs were operationalised, including variability in integration, reliance on remote consultations, and limited protected time. The pandemic legacy may also help explain ongoing workload pressures and the perception of SMRs as an additional demand rather than a fully embedded component of primary care services. As primary care continues to address backlogs and workforce shortages, ensuring that SMRs are supported with adequate infrastructure and protected time is likely to be important for sustainable integration.

Recent NHS England guidance now emphasises the need for patients to understand the purpose of SMRs^39^ and resources have been developed to support clearer communication, including multilingual and accessible materials.^40,41^ However, persistent engagement gaps remain and ongoing work is needed to address understanding and uptake.

Optimising SMRs requires embedding them as routine, holistic reviews and requires clarity of clinical roles, consistent pathways, protected time, and investment in training and professional development. When integrated effectively, SMRs have the potential to identify unmet patient needs, support safe deprescribing to reduce medication-related harm, and improve medicines optimisation, ultimately improving outcomes for people with MLTCs.

These findings should also be considered within a wider and evolving policy landscape. Expansion of clinical pharmacists through the ARRS and the growth of IPs signal a shift towards pharmacists playing a more central role in MLTC management as well as personalised and preventive care. However, future funding arrangements and shifting contractual incentives and priorities may influence how securely SMRs are embedded. Continued evaluation will therefore be important as workforce models and national strategies evolve.

In conclusion, this study highlights the key mechanisms shaping the implementation of SMRs into routine practice in primary care. As NHS England continues to refine national guidance, our findings point to both the promise and the pressures of embedding SMRs sustainably. Optimising SMRs will require strategic and sustained investment from policymakers in training, development and support, clearer definitions of clinical pharmacist roles and responsibilities, and national communication strategies to promote awareness and engagement. These steps could help ensure that SMRs are conducted consistently and meaningfully across primary care settings, offering patients not just a transactional review but a personalised and holistic opportunity to optimise their care.

## References

[1] Parekh N, Ali K, Stevenson JM (2018). Incidence and cost of medication harm in older adults following hospital discharge: a multicentre prospective study in the UK. Brit J Clinical Pharma.

[2] Elliott RA, Camacho E, Jankovic D (2021). Economic analysis of the prevalence and clinical and economic burden of medication error in England. BMJ Qual Saf.

[3] Masnoon N, Shakib S, Kalisch-Ellett L, Caughey GE (2017). What is polypharmacy? A systematic review of definitions. BMC Geriatr.

[4] Guthrie B, Makubate B, Hernandez-Santiago V, Dreischulte T (2015). The rising tide of polypharmacy and drug-drug interactions: population database analysis 1995-2010. BMC Med.

[5] Mangoni AA, Jackson SHD (2004). Age-related changes in pharmacokinetics and pharmacodynamics: basic principles and practical applications. Br J Clin Pharmacol.

[6] Doumat G, Daher D, Itani M (2023). The effect of polypharmacy on healthcare services utilization in older adults with comorbidities: a retrospective cohort study. BMC Prim Care.

[7] Davies LE, Spiers G, Kingston A (2020). Adverse outcomes of polypharmacy in older people: systematic review of reviews. J Am Med Dir Assoc.

[8] Osanlou R, Walker L, Hughes DA (2022). Adverse drug reactions, multimorbidity and polypharmacy. BMJ Open.

[9] Zaninotto P, Huang YT, Gessa G (2020). Polypharmacy is a risk factor for hospital admission due to a fall: evidence from the English Longitudinal Study of Ageing. BMC Public Health.

[10] National Institute for Health and Care Excellence (NICE) (2015). Medicines optimisation: the safe and effective use of medicines to enable the best possible outcomes. NG5. https://www.nice.org.uk/guidance/ng5.

[11] British Medical Association, NHS England (2019). Investment and evolution: a five-year framework for GP contract reform to implement the NHS Long Term Plan. https://www.england.nhs.uk/wp-content/uploads/2019/01/gp-contract-2019.pdf.

[12] NHS England, NHS Improvement (2020). Network Contract Directed Enhanced Service: contract specification 2020/21 – PCN requirements and entitlements. https://www.england.nhs.uk/wp-content/uploads/2020/03/Network-Contract-DES-Specification-PCN-Requirements-and-Entitlements-2020-21-October-FINAL.pdf.

[13] NHS England (2020). Network Contract Directed Enhanced Service. Structured medication reviews and medicines optimisation: guidance. https://www.england.nhs.uk/wp-content/uploads/2020/09/SMR-Spec-Guidance-2020-21-FINAL-.pdf.

[14] NHS England (2024). Network Contract Directed Enhanced Service. Guidance for 2024/25 in England: part A: clinical and support services (section 8). https://www.england.nhs.uk/wp-content/uploads/2024/03/PRN01035_iii_network-contract-DES_part-a-clinical-and-support-services-Section-8-version-3_9-april-2024.pdf.

[15] Duncan P, Cabral C, McCahon D (2019). Efficiency versus thoroughness in medication review: a qualitative interview study in UK primary care. Br J Gen Pract.

[16] Stewart D, Madden M, Davies P (2021). Structured medication reviews: origins, implementation, evidence, and prospects. Br J Gen Pract.

[17] Doherty AS, Boland F, Moriarty F (2023). Adverse drug reactions and associated patient characteristics in older community-dwelling adults: a 6-year prospective cohort study. Br J Gen Pract.

[18] Royal Pharmaceutical Society (2019). Polypharmacy: getting our medicines right.

[19] Picton C, Wright H (2013). Medicines optimisation: helping patients to make the most of medicines.

[20] McCahon D, Denholm RE, Huntley AL (2021). Development of a model of medication review for use in clinical practice: Bristol medication review model. BMC Med.

[21] McEvoy R, Ballini L, Maltoni S (2014). A qualitative systematic review of studies using the normalization process theory to research implementation processes. Implement Sci.

[22] Nuffield Department of Primary Care Health Sciences Optimising StruCtured medicAtion Reviews (OSCAR): a mixed methods process evaluation with integrated real-time observational cohort study. https://www.phc.ox.ac.uk/research/groups-and-centres/hypertension/optimising-structured-medication-reviews-oscar-a-mixed-methods-process-evaluation-with-integrated-real-time-observational-cohort-study.

[23] Sheppard JP, Bateman PA, Wright-Drakesmith C (2025). Impact of structured medication reviews on prescribing in English primary care: a nationwide observational cohort study. medRxiv.

[24] Agwunobi AJ, Seeley AE, Tucker KL (2025). Understanding structured medication reviews delivered by clinical pharmacists in primary care in England: a national cross-sectional survey. BMJ Open.

[25] Saunders B, Sim J, Kingstone T (2018). Saturation in qualitative research: exploring its conceptualization and operationalization. Qual Quant.

[26] May CR, Albers B, Bracher M (2022). Translational framework for implementation evaluation and research: a normalisation process theory coding manual for qualitative research and instrument development. Implement Sci.

[27] May CR, Cummings A, Girling M (2018). Using normalization process theory in feasibility studies and process evaluations of complex healthcare interventions: a systematic review. Implement Sci.

[28] Braun V, Clarke V (2006). Using thematic analysis in psychology. Qual Res Psychol.

[29] Campbell R, Goodman-Williams R, Feeney H (2020). Assessing triangulation across methodologies, methods, and stakeholder groups: the joys, woes, and politics of interpreting convergent and divergent data. Am J Eval.

[30] General Pharmaceutical Council (2023). The GPhC register as of 30 September 2023 – diversity data tables – England. https://assets.pharmacyregulation.org/files/2024-01/gphc-england-register-diversity-data-september-2023_.docx.

[31] Baird B, Lamming L, Bhatt RT (2022). Integrating additional roles into primary care networks. https://assets.kingsfund.org.uk/f/256914/x/1404655eb2/integrating_additional_roles_general_practice_2022.pdf.

[32] Turk A, Wong G, Mahtani KR (2022). Optimising a person-centred approach to stopping medicines in older people with multimorbidity and polypharmacy using the DExTruS framework: a realist review. BMC Med.

[33] Mann C, Anderson A, Boyd M (2022). The role of clinical pharmacists in general practice in England: impact, perspectives, barriers and facilitators. Res Social Adm Pharm.

[34] Anderson C, Zhan K, Boyd M, Mann C (2019). The role of pharmacists in general practice: a realist review. Res Social Adm Pharm.

[35] Bradley F, Seston E, Mannall C, Cutts C (2018). Evolution of the general practice pharmacist’s role in England: a longitudinal study. Br J Gen Pract.

[36] Madden M, Mills T, Atkin K (2022). Early implementation of the structured medication review in England: a qualitative study. Br J Gen Pract.

[37] Swinglehurst D, Hogger L, Fudge N (2023). Negotiating the polypharmacy paradox: a video-reflexive ethnography study of polypharmacy and its practices in primary care. BMJ Qual Saf.

[38] Madden M, Stewart D, Mills T, McCambridge J (2023). Consultation skills development in general practice: findings from a qualitative study of newly recruited and more experienced clinical pharmacists during the COVID-19 pandemic. BMJ Open.

[39] NHS England (2025). Network Contract Directed Enhanced Service. Guidance for 2025/26 in England: part A: clinical and support services (section 8). https://www.england.nhs.uk/wp-content/uploads/2025/03/network-contract-DES-guidance-part-a.docx.

[40] Health Innovation Network Resources to support patients having a structured medication review. https://thehealthinnovationnetwork.co.uk/programmes/medicines/polypharmacy/patient-information.

[41] Howard C, Semple A (2025). Improving access to structured medication reviews in seldom-heard communities: understanding the enablers and barriers to addressing health inequalities. https://cdn.thehealthinnovationnetwork.co.uk/wp-content/uploads/2025/06/Polypharmacy-report-Improving-access-to-SMRs-in-seldom-heard-communities-WEB-2.pdf.

